# Cannabidiol may prevent the development of congestive hepatopathy secondary to right ventricular hypertrophy associated with pulmonary hypertension in rats

**DOI:** 10.1007/s43440-024-00579-4

**Published:** 2024-03-22

**Authors:** Anna Krzyżewska, Marta Baranowska-Kuczko, Anna Galicka, Irena Kasacka, Krzysztof Mińczuk, Hanna Kozłowska

**Affiliations:** 1 Department of Experimental Physiology and Pathophysiology, Medical University of Białystok, Białystok, 15-222 Poland; 2 Department of Clinical Pharmacy, Medical University of Białystok, Białystok, 15-222 Poland; 3 Department of Medical Chemistry, Medical University of Białystok, Białystok, 15-222 Poland; 4 Department of Histology and Cytophysiology, Medical University of Białystok, Białystok, 15-222 Poland

**Keywords:** Congestive hepatopathy, Nuclear factor-κappa B, Inflammation, Cannabidiol, Pulmonary hypertension, Right ventricle

## Abstract

**Background:**

Pulmonary hypertension (PH) can cause right ventricular (RV) failure and subsequent cardiohepatic syndrome referred to as congestive hepatopathy (CH). Passive blood stasis in the liver can affect inflammation, fibrosis, and ultimately cirrhosis. Cannabidiol (CBD) has many beneficial properties including anti-inflammatory and reduces RV systolic pressure and RV hypertrophy in monocrotaline (MCT)-induced PH in rats. Thus, it suggests that CBD may have the potential to limit CH development secondary to RV failure. The present study aimed to determine whether chronic administration of CBD can inhibit the CH secondary to RV hypertrophy associated with MCT-induced PH.

**Methods:**

The experiments involved rats with and without MCT-induced PH. CBD (10 mg/kg) or its vehicle was administered once daily for 3 weeks after MCT injection (60 mg/kg).

**Results:**

Monocrotaline administration increased the liver/body weight ratio. In histology examinations, we observed necrosis and vacuolar degeneration of hepatocytes as well as sinusoidal congestion. In biochemical studies, we observed increased levels of nuclear factor-κappa B (NF-κB), tumour necrosis factor-alpha (TNA-α), interleukin 1 beta (IL-1β), and interleukin 6 (IL-6). CBD administration to PH rats reduced the liver/body weight ratio, improved the architecture of the liver, and inhibited the formation of necrosis. Cannabidiol also decreased the level of NF-κB, TNF-α, IL-1β and IL-6.

**Conclusions:**

The studies show that CBD can protect the liver from CH probably through attenuating PH, protective effects on the RV, and possibly direct anti-inflammatory effects on liver tissue through regulation of the NF-κB pathway.

**Graphical abstract:**

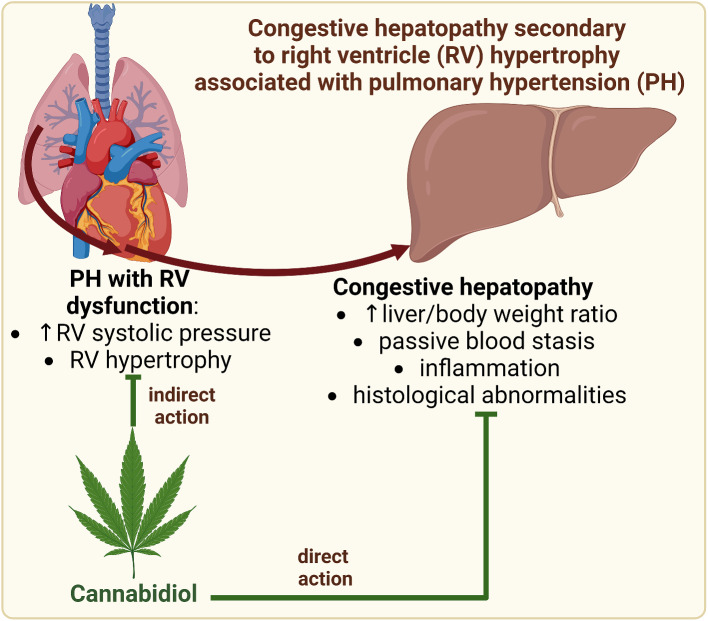

**Supplementary Information:**

The online version contains supplementary material available at 10.1007/s43440-024-00579-4.

## Introduction

Pulmonary hypertension (PH) is a rare disease diagnosed when the mean pressure in the pulmonary artery is higher than 20 mmHg [1]. Under physiological conditions, the right ventricle (RV) is an integral part of the pulmonary circulation, which is characterized by high compliance and low pressure. The RV structure is optimized to adapt to changes in its preload, however, it is less effective in coping with increased afterload caused by high pressure in the pulmonary circulation. In PH, high pressure increases the RV afterload, which consequently contributes to the pathological RV remodeling and its systolic-diastolic failure [2]. Right ventricular failure can lead to cardiohepatic syndrome referred to as congestive hepatopathy (CH). The primary pathomechanism of liver dysfunction is elevated RV pressure transmitted to the liver and portal vein, leading to passive blood stasis which causes the development of CH. It manifests as congestive fibrosis in response to hypoxia, hepatocyte necrosis, venous thrombosis, and inflammation, and can eventually result in cirrhosis [3–6]. Liver damage caused by heart failure is accompanied by passive congestion and activation of Kupffer cells, with the development of inflammation and subsequent activation of multinucleated leukocytes and influx of cytokines and chemokines [7]. The pathophysiology of cardiac-hepatic interactions is not fully understood and it is a subject of intense scientific interest, requiring a well-defined animal model [8]. The animal model of monocrotaline (MCT)-induced PH is one of the most widely used animal models that allow for the development of severe PH, RV hypertrophy, and subsequent CH [6, 9, 10].

Cannabidiol (CBD) is a non-intoxicative phytocannabinoid with many beneficial properties including antioxidant, anti-inflammatory, and anti-fibrotic [11]. It has been demonstrated that CBD, alleviates MCT-induced PH in rats [9, 12] and Sugen + hypoxia-induced PH in mice by reducing RV systolic pressure (RVSP) [12]. In previous publications, we also proved that CBD reduces pulmonary and RV hypertrophy, reduces perivascular and interstitial fibrosis in the RV as well as lowers plasma pro-B-type natriuretic peptide (NT-proBNP) levels in MCT-induced PH [9, 10]. Moreover, CBD reduced fibrosis in the left ventricle in a mouse model of autoimmune myocarditis [13]. This evidence suggests that CBD may have the potential to limit organ changes developing secondary to heart failure (HF) including CH.

The pathomechanism of CH is complex and inflammation may be one of the mechanisms underlying this condition, since activation of hepatic stellate cells (HSCs), a key population of fibrogenic cells in the liver, is an important contributor to the process of liver fibrosis in CH. Studies indicate that the nuclear factor-κappa B (NF-κB) signaling pathway may play a role in liver fibrosis as a potent activator of HSCs [14]. It has been proven that one of the mechanisms of CBD’s anti-inflammatory effects involves the regulation of the NF-κB pathway and reduced production of pro-inflammatory mediators: tumour necrosis factor-alpha (TNF-α), interleukin 1 beta (IL-1β) and interleukin 6 (IL-6) [15]. Recently, it has been suggested that therapeutic targeting of the NF-κB pathway may have beneficial effects in treating liver diseases [16, 17]. Cannabidiol also alleviates non-alcoholic steatohepatitis (NASH) in mice by inhibiting the NF-κB p65 nuclear translocation [17]. In addition to targeting NF-κB, recent reports have shown that a promising therapeutic strategy for PH and its complications may be to inhibit IL-6. The use of a monoclonal antibody (IL-6R Ab) against the interleukin-6 receptor (IL-6R) was effective in reducing elevated pulmonary artery pressure and RVSP, and reduced RV remodeling and inflammation [18]. It is known that one of the anti-inflammatory properties of CBD is the inhibition of IL-6 expression [19], which can directly improve the clinical condition of patients with PH, as well as prevent the development of RV failure and associated CH.

Therefore, this study aimed to determine whether chronic administration of CBD to rats can inhibit the CH secondary to RV hypertrophy associated with MCT-induced PH and whether inflammation is involved in the process.

## Materials and methods

Experiments received approval from the Local Ethical Committee for Animal Experiments at the University of Warmia and Mazury in Olsztyn, Poland (project code 88/2018, granted on November 27, 2018) and were conducted following the ARRIVE guidelines and European Directive (2010/63/EU). Rats were obtained from the Experimental Medicine Centre (Medical University of Białystok, Białystok, Poland). Animals were kept in plastic cages (two rats per cage) under a 12 h/12 h light/dark cycle and had ad libidum access to standard food pellets and water. The experiments were performed on 40 Wistar rats: male, 5–8 weeks old, with body weights: of 150–250 g.

### MCT and CBD treatment

The rats were randomly divided into 4 experimental groups as follows:

1. Control (CTR) rats received solvent for CBD (consisting of Tween 80, ethanol, and 0.9% NaCl in a ratio of 1:3:16) via intraperitoneal (*ip*) administration for 21 days – CTR group.
2. Control rats that received CBD (THC-1073G-1; THC Pharm, Frankfurt, Germany) at a dose of 10 mg/kg for 21 days, (*ip*) – CTR + CBD group.
3. Rats with MCT-induced PH by a single subcutaneous (*sc*) injection of MCT (MCT; C2401-1G; Sigma-Aldrich, Munich, Germany) at a dose of 60 mg/kg and solvent for CBD – MCT group.
4. Rats with MCT-induced PH as in point (3) which were treated with CBD as in point (2) – MCT + CBD.

**Fig. 1:**
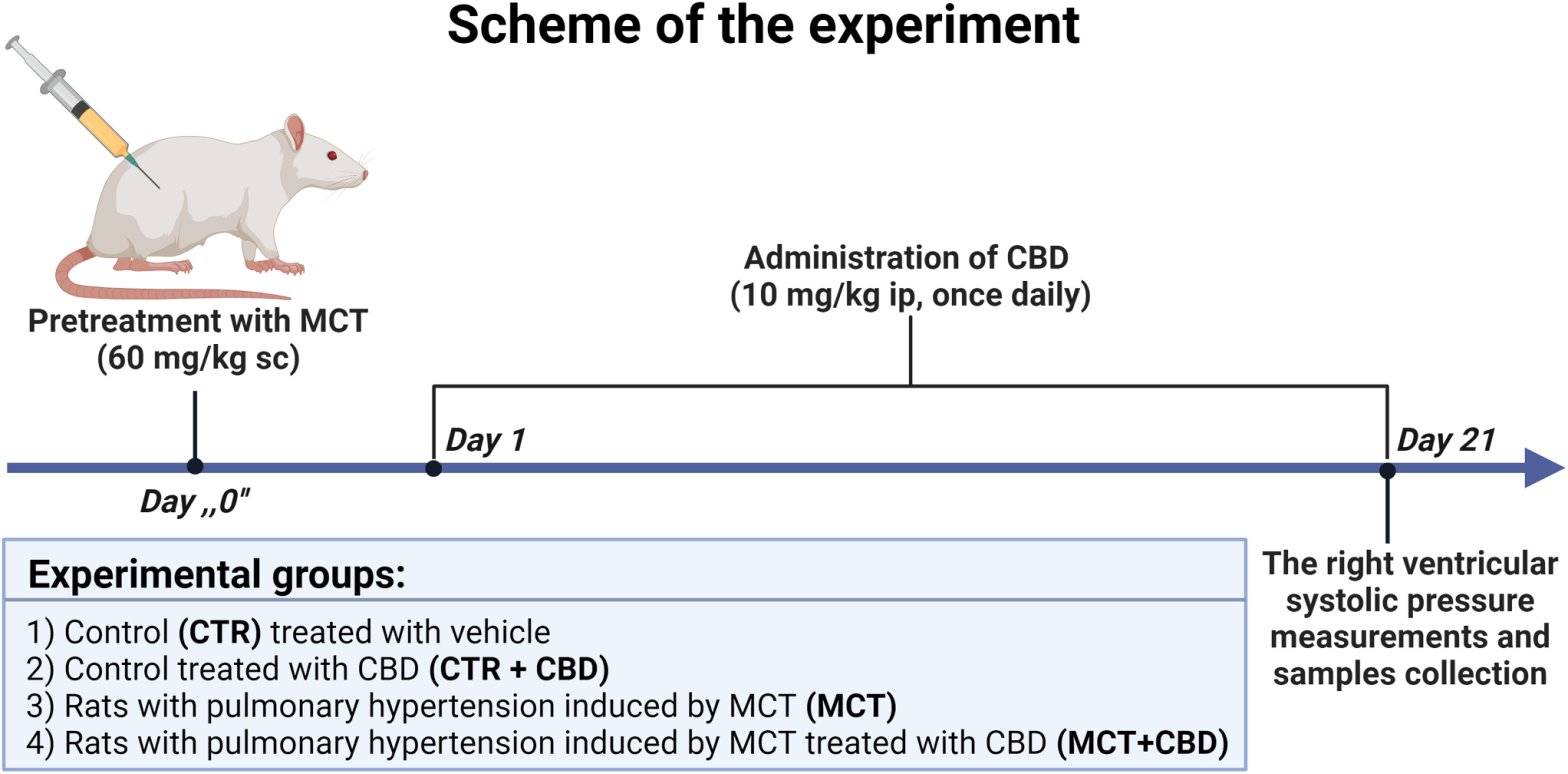
Experimental design. Abbreviations: CBD: cannabidiol; CTR: control; ip: intraperitonially injection; MCT: monocrotaline; sc: subcutaneously injection

The dose of CBD was determined using data, where 10 mg/kg in a rat corresponds to an effective and safe dose of CBD used in humans [20, 21]. PH was confirmed in our previous paper [9] where the RVSP in the MCT group was 43.7 ± 3.9, in the CTR group 20.03 ± 0.9, in CTR + CBD 21.4 ± 0.9 and MCT + CBD 28.2 ± 0.7 [mmHg] (*n* = 10). Figure 1 shows the protocol of the experiments.

### Plasma parameters evaluation

Total bilirubin, albumin, aspartate transaminase (AST), alanine transaminase (ALT), glutamate dehydrogenase (GLDH), and gamma-glutamyltransferase (GGT) activity were determined on a Cobas 6000 (Hitachi, Tokyo, Japan).

### Tissue preparation

The animals underwent anesthesia (sodium pentobarbital (300 µmol/kg), Biowet, Puławy, Poland) a day after the last administration of CBD. The parts of the liver tissues were destined for histology studies (described below). The remains of liver tissue were weighed and then immediately frozen (flash frozen) with liquid nitrogen and stored at − 80 °C for Western blot, enzyme-linked immunosorbent assay (ELISA), and quantitative Real-Time PCR (qRT-PCR) studies.

### Histology

Liver sections were fixed in 10% buffered formalin, cut into small pieces, put in a tissue cassette for the dehydration process, embedded in paraffin, sliced (4 μm), and stained with hematoxylin and eosin (H + E). Histopathological changes of each liver section (5 sections per each animal (*n* = 5–6)) were blindly evaluated in ≥ 10 randomly selected non-overlapping fields using an Olympus BX43 (Olympus 114 Corp., Tokyo, Japan) microscope equipped with an Olympus DP12 camera at 200× magnifications. In each liver sample, the percentage area of sinusoidal congestion, necrosis, and vacuolization was assessed with the ImageJ software version 1.53c (NIH, Bethesda, MD, USA) using Suzuki’s classification according to the following criteria [22]:

- 0 score – no congestion, necrosis, vacuolization (%),
- 1 score – very slight changes (1–10%) in congestion and vacuolization and single cells necrosis,
- 2 score – slight changes (11–30%) in congestion and vacuolization and < 30% of necrosis cells,
- 3 score – moderate changes (31–60%) in congestion, vacuolization, and necrosis,
- 4 score – severe changes (> 60%) in congestion, vacuolization, and necrosis.

### Western blot

Liver samples were homogenized in a buffer Mammalian Protein Extraction Reagent (MPER, Thermo Scientific, Rockford, IL, USA) containing a protease inhibitor cocktail (Roche Diagnostics GmbH, Germany). The bicinchoninic acid method (Price Rapid Gold BCA, Protein Assay Kit, Thermo Scientific, Waltham, MA, USA) was used to determine the total protein concentration, and after calculation homogenates were properly reconstituted in Laemmli buffer (Bio-Rad Laboratories, Inc., Hercules, CA, USA). Following, electrophoresis (200 V, 1 h) and transfer (100 V, 45 min). Next, the membranes underwent overnight incubation at 4 °C with specific primary antibodies: NF-κB (1:1000; sc-514,451, Santa Cruz Biotechnology, Dallas, TX, USA), CB_1_-R (1:500; ab259323, Abcam, Cambridge, UK), CB_2_-R (1:500; ab3561, Abcam, Cambridge, UK), and GAPDH (1:20000; EPR16891, Abcam, Cambridge, UK) and then incubation with secondary antibodies in room temperature for 2 h (1:3000; ab6721, Abcam, Cambridge, UK). The protein bands were measured densitometrically with a ChemiDoc visualization system (Image Laboratory Software Version 6.0.1; Bio-Rad, Warsaw, Poland) and normalized to GAPDH. The level of protein was calculated using ImageJ software version 1.53c (NIH, Bethesda, MD, USA).

### ELISA method

The concentrations of TNF-α and IL-1β (ab100785 and ab255730; Abcam, Cambridge, UK) were determined through enzyme-linked immunosorbent assay (ELISA), following the guidelines provided by the manufacturer.

### qRT-PCR

The liver was homogenized in TRIsure™ (BIO-38,032, Bioline GmbH, Luckenwalde, Germany) and RNA was isolated following the guidelines provided by the manufacturer. RNA was reverse-transcribed into cDNA using SensiFAST™ cDNA Synthesis Kit (BIO-65,053, Bioline, London, UK). Quantitative Real-Time PCR was performed in a thermocycler (Thermocycler CFX96 Real-Time System, Bio-Rad) using a SensiFAST™ SYBR Kit (BIO-98,005, Bioline). The sequence of the primers and parameters of qRT-PCR are presented in Table 1 (provided by Genomed, Warsaw, Poland). Relative expression was calculated using the delta–delta Cycle Threshold (Ct) method and normalized to *GAPDH* [23].

**Table 1:** Sequence and hybridization temperature of specific primers

Gene	Forward primer (5′→3′)	Reverse primer (5′→ 3′)	T (°C)
*TGF-β*	TGC TAA TGG TGG ACC GCA A	CAC TGC TTC CCG AAT GTC TGA	60
*COL1A1*	ATC AGC CCA AAC CCC AAG GAG A	CGC AGG AAG GTC AGC TGG ATA G	60
*TNF-α*	ACT GAA CTT CGG GGT GAT TG	GCT TGG TGG TTT GCT ACG AC	58
*IL-6*	TGA TGG ATG CTT CCA AAC TG	GAG CAT TGG AAG TTG GGG TA	57
*GAPDH*	CCA TTC TTC CAC CTT TGA TGC T	TGT TGC TGT AGC CAT ATT CAT TGT	60

*Abbreviations* COL1A1: collagen I; GAPDH: glyceraldehyde 3-phosphate dehydrogenase; IL-6: interleukin 6; TGF-β1: transforming growth factor beta 1; TNF-α: tumour necrosis factor-alpha

### Statistical analysis

Statistical analysis was performed using GraphPad Prism 9.3.0 (GraphPad Software, San Diego, California, USA). The normality of the data was assessed for Gaussian distribution before conducting statistical tests. The data exhibited a normal distribution a two-way analysis of variance (ANOVA) with Tukey’s multiple comparisons test (when appropriate) was used. The results are presented as mean ± the mean standard error of the mean (SEM) of n animals. For non-parametric data (scores), a non-parametric Kruskal-Wallis test followed by post-hoc Dunn’s (when appropriate) test was used and the data was expressed as medians and interquartile ranges. A significance level of p–value < 0.05 was considered statistically significant.

## Results

### Effects of MCT-induced PH and CBD on liver/total body weight ratio and selected liver function parameters in plasma

We observed the statistically significant effect of MCT (MCT effect: F_1,36_ = 19.06, *p* < 0.001) and CBD (CBD effect: F_1,36_ = 10.22, *p* = 0.003) on the liver/total body weight ratio in groups with the interaction between factors i.e., MCT x CBD (F_1,36_ = 11.40, *p* = 0.002). Monocrotaline rats showed a higher liver/total body weight ratio of about 20% relative to CTR (Tukey multiple comparison test: q = 7.743, DF = 36, *p* < 0.001) rats suggesting CH. Chronic CBD administration to MCT rats reduced this ratio by 13% (Tukey multiple comparison test, q = 6.574, DF = 36, *p* < 0.001) (Table 2).

**Table 2:** Effects of cannabidiol on liver/total body weight ratio and selected liver function parameters in plasma of control and monocrotaline-induced pulmonary hypertension rats

Parameter	n	CTR	CTR + CBD	MCT	MCT + CBD	F and p-value
Liver/body weight ratio	6	35.0 ± 1.0	34.6 ± 0.7	42.8 ± 1.3***	37.2 ± 1.2^###^	MCT effect: F_1,36_ = 19.06, *p* < 0.001 CBD effect: F_1,36_ = 10.22, *p* = 0.003 Interaction (MCT × CBD): F_1,36_ = 11.40, *p* = 0.002
ALT (U/l)	6	23.3 ± 5.0	21.8 ± 3.8	22.5 ± 3.1	20.2 ± 4.6	MCT effect: F_1, 16_ = 0.1500, *p* = 0.704 (NS); CBD effect: F_1, 16_ = 3.267, *p* = 0.090 (NS); Interaction (MCT × CBD): F _1, 16_ = 1.350, *p* = 0.262 (NS)
AST (U/l)	6	65.0 ± 26.6	63.8 ± 20.3	49.5 ± 9.1	48.7 ± 8.4	MCT effect: F_1, 18_ = 2.494, *p* = 0.132 (NS); CBD effect: F_1, 18_ = 0.1168, *p* = 0.736 (NS); Interaction (MCT × CBD): F _1, 18_ = 0.2721, *p* = 0.608 (NS)
Albumin (g/dl)	6	3.5 ± 0.3	3.2 ± 0.3	3.2 ± 0.2	3.0 ± 0.3	MCT effect: F_1, 18_ = 3.740, *p* = 0.069 (NS); CBD effect: F_1, 18_ = 1.195, *p* = 0.289 (NS); Interaction (MCT × CBD): F _1, 18_ = 3.740, *p* = 0.703 (NS)
Total bilirubin (mg/dl)	6	< 0.15	< 0.15	< 0.15	< 0.15	–
GLDH (U/l)	6	4.5 ± 0.8	4.2 ± 1.5	4.7 ± 1.0	3.8 ± 1.2	MCT effect: F_1, 20_ = 0.031, *p* = 0.861 (NS); CBD effect: F_1, 20_ = 1.541, *p* = 0.229 (NS); Interaction (MCT × CBD): F _1, 20_ = 0.283, *p* = 0.601 (NS)
GGT (U/l)	6	< 3	< 3	< 3	< 3	–

Cannabidiol (CBD) or its vehicle was administrated once a day for 21 days. Data are presented as mean ± SEM; *n* = 5–6 animals per group. Two-way ANOVA, followed by Tukey’s multiple comparisons test. A significance level is set at *p* < 0.05. In total bilirubin and gamma-glutamyltransferase results are under the detection threshold thus the statistic comparison is impossible. *** *p* < 0.001 CTR vs. MCT, ^###^*p* < 0.001 MCT vs. MCT + CBD. Abbreviations: ALT: alanine transaminase; AST: aspartate transaminase; CTR: control; GGT: gamma-glutamyltransferase; GLDH: glutamate dehydrogenase; MCT: monocrotaline; NS: nonspecific

Analysis of plasma ALT, AST, albumin, total bilirubin, GLDH, and GGT parameters showed no differences between CTR and MCT rats suggesting no damage to hepatocytes. Chronic administration of CBD did not modify these parameters in either the CTR + CBD or MCT + CBD groups (Table 2), for statistical significance, see Table 2.

### Effects of MCT-induced PH and CBD on sinusoidal congestion, vacuolization of hepatocyte cytoplasm, and necrosis evaluated according to Suzuki’s classification

**Fig. 2:**
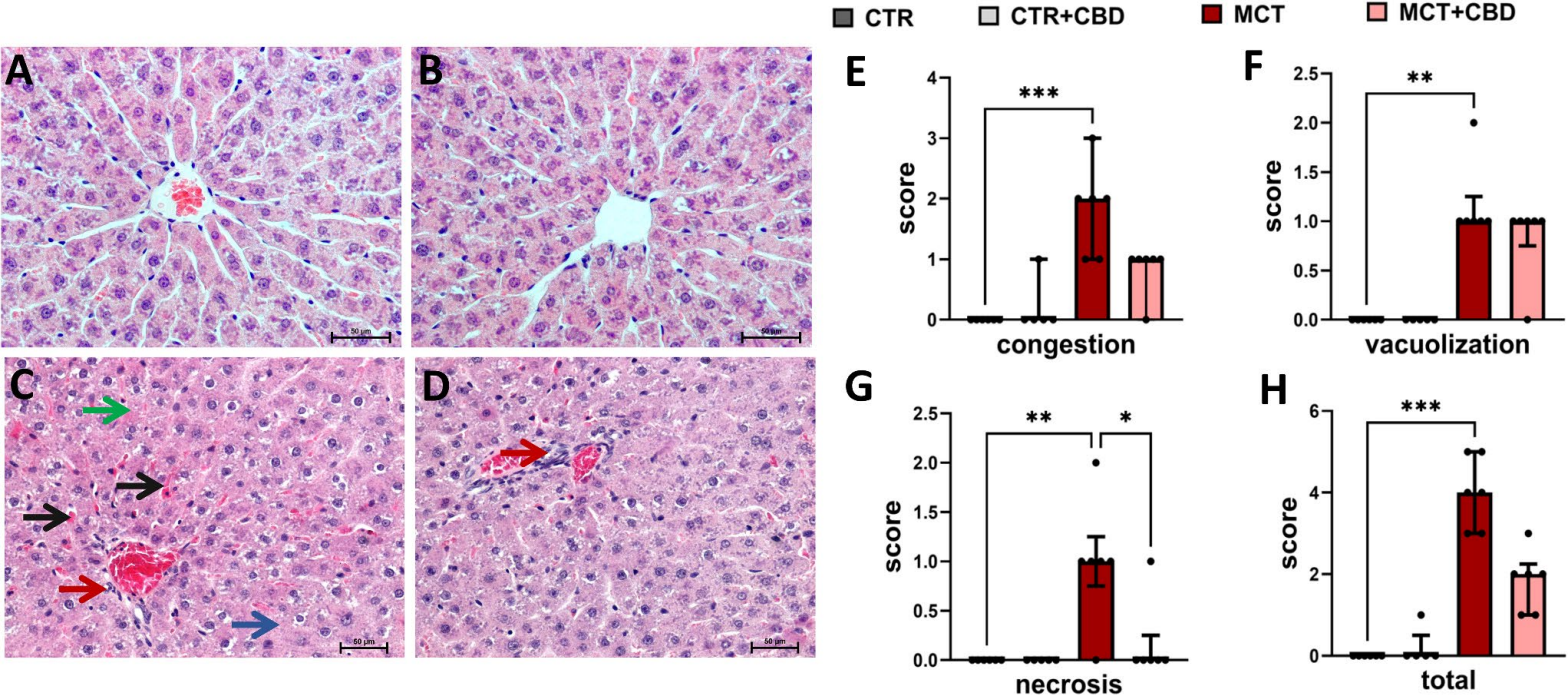
Representative liver sections stained with hematoxylin and eosin control (CTR) rats (**A**), CTR rats treated with cannabidiol (CBD) (**B**), rats with monocrotaline (MCT)-induced pulmonary hypertension (**C**), MCT rats treated with CBD (**D**). The *black arrows* indicate congestion, the *blue arrow* indicates the area of necrosis, the *green arrow* indicates vacuolization and the *red arrows* indicate the influx of inflammatory cells around the central vein. Magnification ×200 (Olympus BX43 (Olympus 114 Corp., Tokyo, Japan)); scale bar = 50 μm. Quantification of liver congestion (**E**), hepatocyte vacuolization (**F**), necrosis (**G**), and total changes in the scores above-mentioned liver pathologies (**H**) a according to the Suzuki’s classification (0–4 score). Data are presented as medians and interquartile ranges; *n* = 5–6 animals per group. The Kruskal-Wallis followed by post-hoc Dunn’s test. A significance level is set at *p* < 0.05

We observed the statistically significant increase of congestion score (Kruskal–Wallis followed by post-hoc Dunn’s test (H = 16.91, N1 = 6, N2 = 5, N3 = 6, N4 = 6; *p* < 0.001, CTR vs. MCT, z = 3.744, *p* < 0.001)), vacuolization score (Kruskal–Wallis followed by post-hoc Dunn’s test (H = 18.47, N1 = 6, N2 = 5, N3 = 6, N4 = 6; *p* < 0.001, CTR vs. MCT, z = 3.452, *p* = 0.002)), necrosis score (Kruskal-Wallis followed by post-hoc Dunn’s test (H = 13.85, N1 = 6, N2 = 5, N3 = 6, N4 = 6; *p* = 0.003, CTR vs. MCT, z = 3.222, *p* = 0.004)), and total score (Kruskal–Wallis followed by post-hoc Dunn’s test (H = 20.10, N1 = 6, N2 = 5, N3 = 6, N4 = 6; *p* < 0.001, CTR vs. MCT, z = 3.968, *p* < 0.001)) in MCT group of rats compared to CTR group (Fig. 2).

Chronic CBD administration to MCT rats reduced only the score of necrosis Kruskal–Wallis followed by post-hoc Dunn’s test (H = 13.85, N1 = 6, N2 = 5, N3 = 6, N4 = 6; *p* = 0.003, MCT vs. MCT + CBD, z = 2.611, *p* = 0.027)). In congestion and total score we observed only a decreasing tendency (Fig. 2).

### Effects of MCT-induced PH and CBD on selected parameters of inflammation and fibrosis

**Fig. 3:**
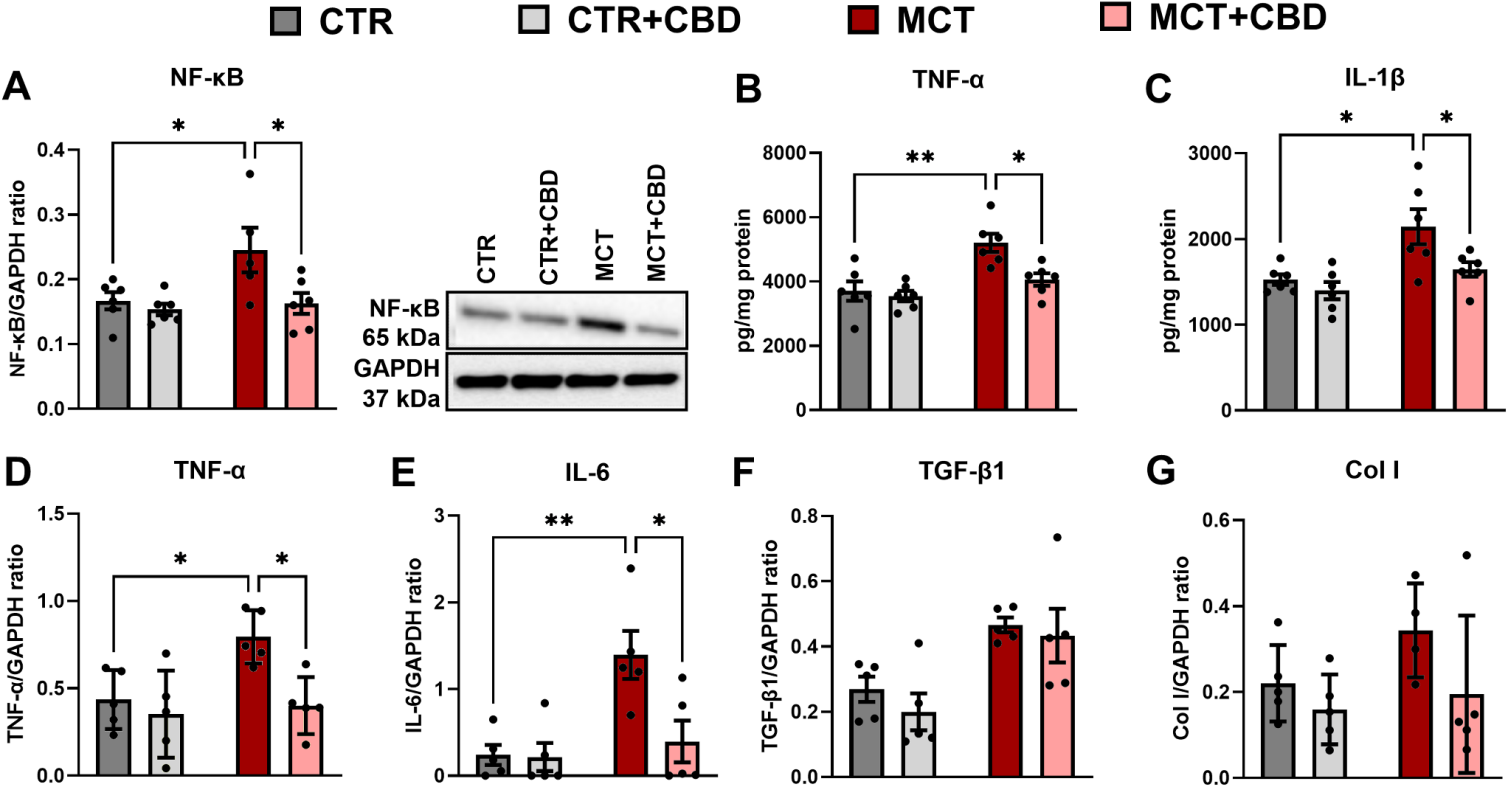
The influence of cannabidiol (CBD) on the protein level of nuclear factor-κappa B (NF-κB) determined by Western blot (**A**); the concentration of tumour necrosis factor-alpha (TNF-α) (**B**) and interleukin 1 beta (IL-1β) (**C**) determined by enzyme-linked immunosorbent assay (ELISA) method; the mRNA expression level of TNF-α (**D**), interleukin 6 (IL-6) (**E**), transforming growth factor-beta 1 (TGF-β1) (**F**), and collagen type I (Col I) (**G**), in livers from control (CTR) and monocrotaline (MCT)-induced pulmonary hypertension rats. All data are presented as mean ± SEM, *n* = 5–6; *p < 0.05, **p < 0.01, ***p < 0.001; a two-way ANOVA followed by Tukey’s post-hoc test

We observed the influence of MCT (MCT effect: F_1, 19_ = 5.319, *p* = 0.033) and CBD (CBD effect: F_1, 19_ = 6.369, *p* = 0.021) on the expression of NF-κB in groups with no interaction between factors (F_1, 19_ = 3.316, *p* = 0.084). In the MCT group, the expression of NF-κB was increased by about 50% compared to the CTR group (Tukey multiple comparison test: q = 4.032, DF = 19, *p* = 0.046), and chronic CBD administration to MCT rats resulted in a decrease in the expression of NF-κB by about 30% compared to the MCT group (Tukey multiple comparison test: q = 4.245, DF = 19, *p* < 0.034) (Fig. 3A). We observed influence of MCT (MCT effect: F_1, 20_ = 17.09, *p* < 0.001 for TNF-α and MCT effect: F_1, 20_ = 11.88, *p* = 0.003 for IL-1β) and CBD (CBD effect: F_1, 20_ = 7.151, *p* = 0.015 for TNF-α and CBD effect: F_1, 20_ = 6.247, *p* = 0.021 for IL-1β) on the expression of TNF-α and IL-1β in groups with no interaction between factors in both cases (for TNF- α: F_1, 20_ = 4.115, *p* = 0.056 and for IL-1β: F_1, 20_ = 2.179, *p* = 0.155). In the MCT group, we observed increased concentration of TNF-α and IL-1β (by about 40% in both cases) in the liver tissue compared to the CTR group (Tukey multiple comparison test: q = 6.163, DF = 20, *p* = 0.002 for TNF-α and q = 4.923, DF = 20, *p* = 0.012 for IL-1β). Chronic administration of CBD to MCT rats decreased the concentration of TNF-α and IL-1β (by about 20% in both cases) compared to the MCT group (Tukey multiple comparison test: q = 4.703, DF = 20, *p* = 0.016 for TNF-α and q = 3.976, DF = 20, *p* = 0.049 for IL-1β) (Fig. 3B and C). The TNF-α, IL-6, transforming growth factor beta 1 (TGF-β1), and collagen I (Col I), expression in liver tissue was determined by qRT-PCR method (Fig. 3). The direction of changes in the TNF-α parameter was the same at both the mRNA and protein levels, therefore we decided to evaluate the rest of the inflammatory parameters already at the protein level. We observed influence of MCT (MCT effect: F_1, 16_ = 5.920, *p* = 0.027 for TNF-α and MCT effect: F_1, 16_ = 10.21, *p* = 0.006 for IL-6) and CBD (CBD effect: F_1, 16_ = 8.081, *p* = 0.012 for TNF-α and CBD effect: F_1, 16_ = 6.005, *p* = 0.026 for IL-6) on the expression of TNF-α and IL-6 in groups with no interaction between factors for TNF- α (F_1, 16_ = 3.426, *p* = 0.083) and with interaction between factors for IL-6 (F_1, 16_ = 5.458, *p* = 0.033). In the livers of the MCT group, the expression of TNF-α and IL-6 was increased by about 80% and 5-fold, respectively compared to the CTR group (Tukey multiple comparison test: q = 4.284, DF = 16, *p* = 0.036 for TNF-α and q = 5.532, DF = 16, *p* = 0.006 for IL-6), and chronic CBD administration to MCT rats resulted in a decrease in the expression of TNF-α and IL-6 by about 50% and 60%, respectively compared to the MCT group (Tukey multiple comparison test: q = 4.694, DF = 16, *p* = 0.020 for TNF-α and q = 4.787, DF = 16, *p* = 0.018 for IL-6) (Fig. 3D, E).

We observed influence of MCT (MCT effect: F_1, 16_ = 15.55, *p* = 0.01) but no CBD (CBD effect: F_1, 16_ = 0.8653, *p* = 0.366) on the expression of TGF-β1 in groups with no interaction between factors F_1, 16_ = 0.1104, *p* = 0.774. Tukey multiple comparison test showed differences between the CTR + CBD vs. MCT (q = 4.873, DF = 16, *p* = 0.016) and CTR + CBD vs. MCT + CBD (q = 4.275, DF = 16, *p* = 0.037) groups, however, in the context of our study, significances in these groups were not considered and are not shown in Fig. 3. The expression of Col I (Fig. 3G) was at a similar level in each group (no MCT effect: F_1,15_ = 1.934, *p* = 0.185, no CBD effect: F_1,15_ = 3.385, *p* = 0.086, no interaction between factors: F_1,15_ = 0.5934, *p* = 0.453) but we noticed an increasing tendency in the expression of this parameter in the MCT group and a decreasing tendency after CBD administration to MCT rats (MCT + CBD) (Fig. 3G).

### Effects of MCT-induced PH and CBD on the expression of cannabinoid receptors

**Fig. 4:**
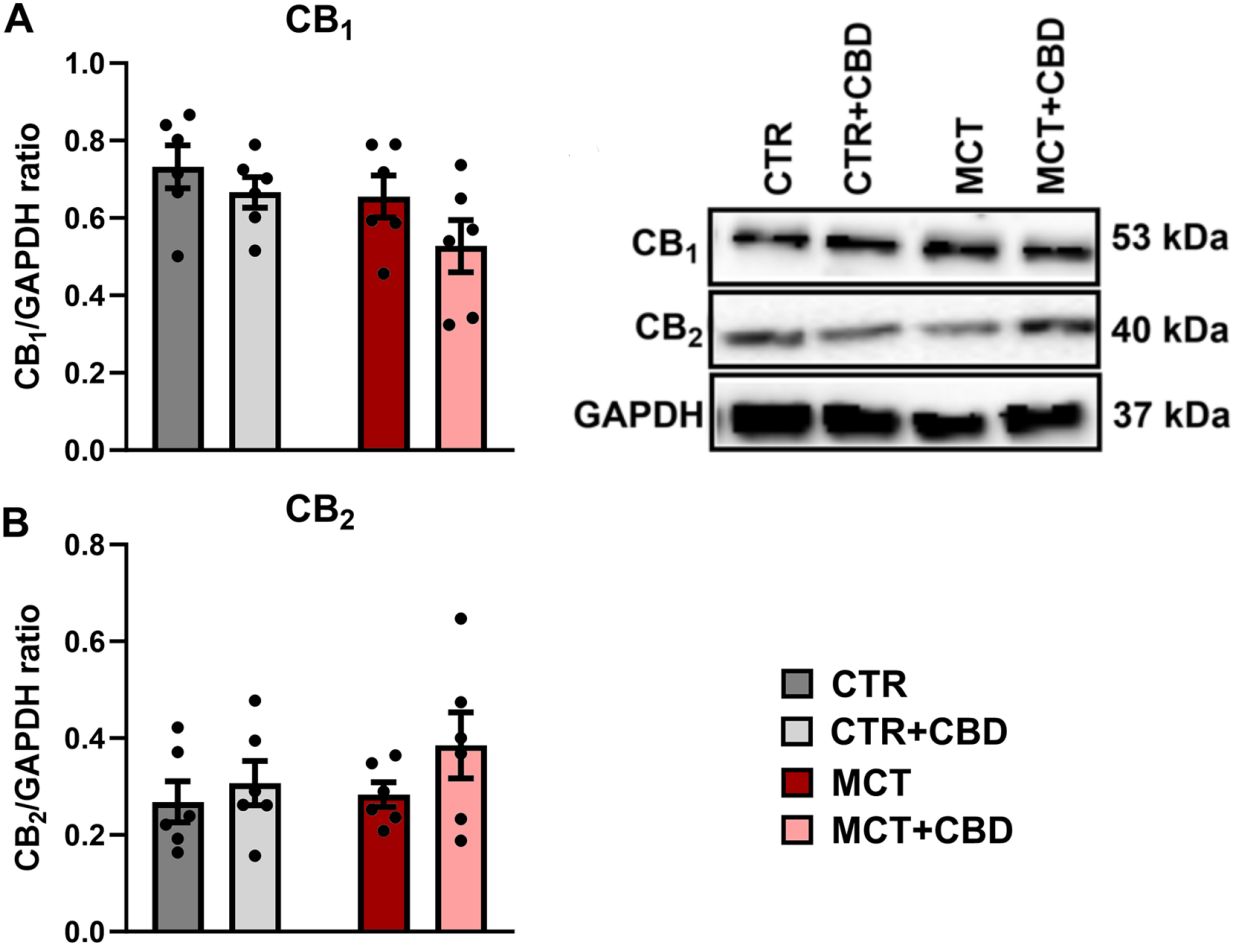
The influence of cannabidiol (CBD) on the protein levels of cannabinoid type 1 receptor (CB_1_) (**A**), cannabinoid type 2 receptor (CB_2_) (**B**) determined by Western blot in livers from control (CTR) and monocrotaline (MCT)- induced pulmonary hypertension rats. All data are presented as mean ± SEM, *n* = 6; a two-way ANOVA followed by Tukey’s post-hoc test

Western blot analysis showed that the expressions of cannabinoid receptors 1 and 2 (CB_1_-Rs and CB_2_-Rs) were at a similar level in each group (no MCT effect: F_1,20_ = 3.828, *p* = 0.064, no CBD effect: F_1,20_ = 3.112, *p* = 0.093, no interaction between factors: F_1,20_ = 0.3277, *p* = 0.573 for CB_1_-Rs and no MCT effect: F_1,20_ = 0.9380, *p* = 0.344, no CBD effect: F_1,20_ = 2.151, *p* = 0.158, no interaction between factors: F_1,20_ = 0.4325, *p* = 0.518 for CB_2_-Rs) (Fig. 4).

## Discussion

In this study, MCT administration increased the liver/body weight ratio, necrosis and vacuolar degeneration of hepatocytes, as well as sinusoidal congestion. In biochemical studies, we also observed increased levels of NF-κB, TNA-α, IL-1β, and IL-6. CBD administration to PH rats reduced the liver/body weight ratio and inhibited the formation of necrosis. Cannabidiol also decreased the level of NF-κB, TNF-α, IL-1β and IL-6.

In our experiments, despite the lack of effect of MCT at 60 mg/kg on plasma parameters, the ratio of liver/body weight increased 21 days after MCT administration. It may indicate the onset of CH development in the MCT group due to passive blood stasis in the liver caused by RV failure rather than toxic MCT-induced liver damage. Our previous papers support these speculations as using the same rats, we have demonstrated PH with RV failure as evidenced by increased RVSP, elevated Fulton index (the RV weight to the left ventricle (LV) plus septum (RV/LV + S) ratio) [9], as well as cardiomyocyte enlargement and RV fibrosis [10]. Dai et al. [3] also did not show an increase in ALT, AST, GGT, and total bilirubin in the serum of patients with CH. Similarly, a single oral administration of MCT at doses of 40, and 80 mg/kg did not modify the activity of liver enzymes AST and ALT in rat plasma, only 240 mg/kg increased the activity of the aforementioned enzymes [24]. Otaka et al. [25] demonstrated that a single administration of MCT at a dose of 600 mg/kg to mice resulted in higher ALT activity, peaking at 48 h after injection and returning to normal after 120 h. The above reports and the fact that the liver has a high regenerative potential suggest that only high doses of MCT cause toxic damage to the liver and regeneration occurs after 120 h [25].

We showed neither beneficial nor adverse effects of CBD administration on plasma parameters. Still, we observed prevention of liver enlargement in the MCT + CBD group compared to MCT rats, presumably through the beneficial effect on RV function that we have already demonstrated in our previous studies [9, 10]. Thus, we confirmed the results of other authors where chronic (90 days) oral administration of CBD at doses of 0.66 and 1.33 mg/kg to healthy rats did not modify the most biochemical parameters (e.g., AST, ALT) and did not modify liver weight or liver/body weight ratio [26]. It correlated with no abnormalities in hepatic morphology in the CTR and CTR + CBD groups (Fig. 2A, B).

The histological images confirm the development of mild sinusoidal congestion 21 days after MCT injection (Fig. 2C, E). Based on the pathological changes in the liver described in the results section we suggest that during passive venous congestion, hepatic cells became ischemic with the initiation of metabolic changes leading to accumulation of sodium and water in the cell. Gewehr et al. [6] showed that a single administration of 60 mg/kg MCT to rats causes CH secondary to PH-related RV hypertrophy. After 15, 30, and 37 days after MCT administration, histological changes were found in the liver of these animals. After 15 days, mild sinusoidal hyperemia was observed, which intensified to severe on the 37th day of the study [6]. The limitation of our study is that it lasted only 21 days. Prolonged exposure of the liver to passive congestion could have induced fibrotic changes and would have allowed us to assess the effect of CBD on more advanced changes typical of CH. However, prolonging the study was associated with increased mortality in rats due to severe PH. In the future, we plan to study liver function in a different PH model, the use of which may allow us to extend the experiment.

Accordingly, we are the first to prove that CBD can alleviate CH secondary to RV hypertrophy associated with PH. We suggest that one of the likely mechanisms of hepatoprotective effects involves the improvement of cardiac function by chronic administration of CBD. In our previous papers, we already proved that CBD reduces perivascular and interstitial fibrosis in the RV, as well as decreases cardiomyocyte width [10] which was associated with lower RV systolic pressure [9] and may have contributed to the inhibition of the development of mild sinusoidal congestion.

In PH rats, histological examination showed the beginning of the development of sinusoidal congestion and vacuolization of hepatocyte cytoplasm. We did not observe advanced fibrosis, only an upward trend in TGF-β1 and Col I parameters, which are among the markers of remodeling and fibrosis. Interestingly, before the development of fibrotic lesions, we observed in PH rats an activation of the NF-κB pathway, which regulates the secretion of the mediators TNF-α, IL-1β and IL-6, and in histological images we observed an influx of inflammatory cells in the area of the central vein. There is still a lot of ambiguity about the mechanisms of CH development which is due to the lack of an adequate experimental model to determine the molecular mechanisms contributing to the development of CH [5]. As mentioned, inflammation in the course of CH which has been overlooked for many years, has recently become the focus of research. In liver damage caused by heart failure, concomitant passive congestion is accompanied by activation of Kupffer cells, with subsequent activation of multinucleated leukocytes and influx of cytokines and chemokines [7]. Hamberger et al. [14] observed that the livers of rats with PH induced by Sugen + hypoxia developed congestive liver failure secondary to HF, and higher expression of T lymphocytes, macrophages, and increased expression of TNF-α were found. In patients with CH, moderate hepatitis with mild liver cell damage has been observed [3]. Hamberger et al. [14] showed that activation of HSCs, part of the main fibrogenic cell population in the liver, contributes to hepatic fibrosis in CH. Importantly, the NF-κB signaling pathway is a potent activator of HSC and thus may be the mechanism that initiates fibrosis in CH [27]. It is known that fibrotic changes occur in advanced CH [7]. One of the central regulators of fibrosis that leads to deposition of extracellular matrix and production of, for example, collagen I in many liver diseases of various etiologies is TGF-β1 [28]. Overexpression of TGF-β1 leads to activation and migration of HSCs, which as mentioned above are responsible for fibrotic changes in CH [5]. Known factors leading to increased TGF-β1 expression are IL-1β and also NF-κB [29]. In our study, we observed an increase in both NF-κB and IL-1β in the livers of rats after MCT administration, as well as a trend toward an increase in TGF- β1, suggesting that rats on day 21 after MCT administration had already developed inflammation in the liver, but fibrosis processes had not yet been initiated, which is also confirmed by histological images (Fig. 2C).

Cannabidiol in our study decreased the level of NF-κB, TNF-α, IL-6, and IL-1β parameters, suggesting that it may inhibit the NF-κB pathway and thus regulate inflammation in the liver. NF-κB is assumed to be a central regulator of inflammation that responds to a wide variety of immune receptors, and its dysregulated activation has been linked to many inflammatory diseases. It is reported that targeting the NF-κB pathway may find application in the treatment of inflammatory diseases [30]. Previously, we confirmed that CBD can regulate the activity of the NF-κB pathway in the lungs of rats with MCT-induced PH [31]. In addition, CBD in the livers of mice with NASH reduced the translocation of NF-κBp65 which leads to a decrease in the expression of TNF-α and IL-1β. The same authors suggest that inactivation of NF-κB in macrophages by CBD may be a key mechanism for its anti-inflammatory effects in the liver [17]. Moreover, CBD administration inhibited macrophage recruitment and suppressed the activation of the NF-κB pathway in the livers of mice with NASH thus confirming the hepatoprotective properties of CBD [32]. We suggest that CBD, through its direct effect on inhibiting the NF-κB pathway, may indirectly contribute to inhibiting the activation of HSCs and thus prevent liver fibrosis, the next stage of CH. Other researchers have shown that CBD, by inducing the selective death of activated HSCs, may find use as a therapeutic agent for liver fibrosis [33]. Effects of CBD on other liver cell types have also been reported. CBD inhibited NF-κB activation and TNF-α production in response to endotoxin in mouse Kupffer cells and reduced the expression of adhesion molecules and multinucleated cell adhesion to human sinusoidal endothelial cells [34].

Since there have been reports that the activation of CB_1_-Rs has a fibrogenesis-promoting effect on the liver, and activation of CB_2_-Rs has a hepatoprotective effect [35], we tested whether PH and administration of CBD affect the expression of classical cannabinoid receptors. We noted no effect of MCT or CBD on the expression of these receptors. This is not surprising, since CBD has a low affinity for cannabinoid receptors [21] and its hepatoprotective effect probably includes other mechanisms.

## Conclusions

**Fig. 5:**
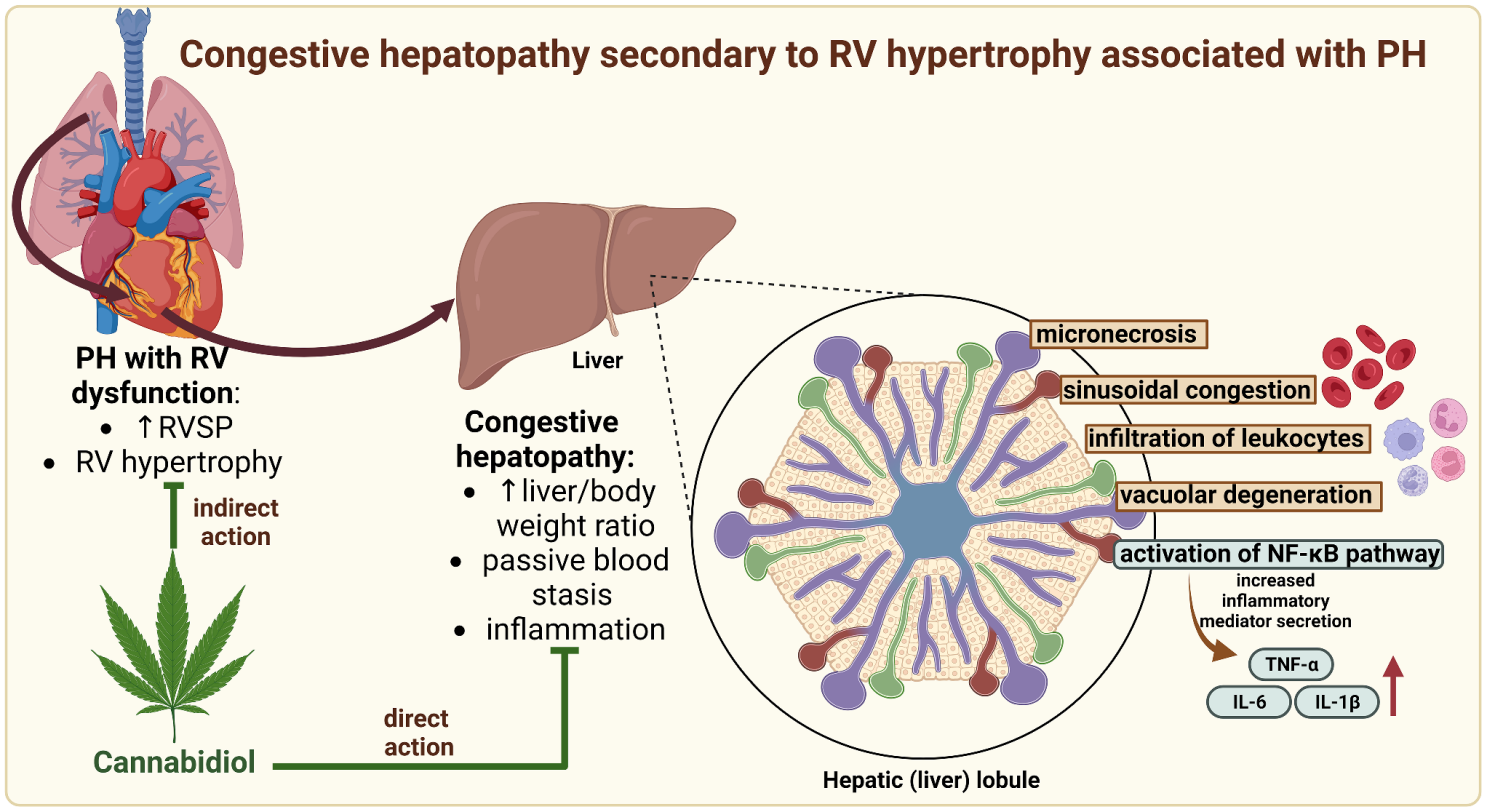
The proposed hepatoprotective effect of cannabidiol in congestive hepatopathy is secondary to right ventricular hypertrophy associated with pulmonary hypertension. Abbreviations: IL-1β: interleukin 1 beta; IL-6: interleukin 6; NF-κB: nuclear factor-κappa B; PH: pulmonary hypertension; RV: right ventricle; RVSP: right ventricular systolic pressure; TNF-α: tumour necrosis factor alpha. Created in Biorender.com

In conclusion, we confirmed that the NF-κB pathway may be involved in the development of CH, especially at an early stage. Furthermore, the studies presented show that CBD can protect the liver from CH probably through attenuating PH [9], protective effects on the RV [10], and possibly direct anti-inflammatory effects on liver tissue through regulation of the NF-κB pathway (Fig. 5). In addition, like other authors, we confirm that CBD did not cause any adverse changes in the liver of healthy rats, demonstrating its high safety potential.

### Electronic supplementary material

Below is the link to the electronic supplementary material.

Supplementary Material 1

## Data Availability

Available from the corresponding author upon reasonable request.
