# Supplementary material for: Cannabidiol may prevent the development of congestive hepatopathy secondary to right ventricular hypertrophy associated with pulmonary hypertension in rats
Source: Pharmacol Rep. 2024 Mar 22;76(2):424–34. doi: 10.1007/s43440-024-00579-4 (PMC11016513; doi:10.1007/s43440-024-00579-4)
Supplement: Supplementary file 1 — Supplementary Material 1 [file 43440_2024_579_MOESM1_ESM.pdf]

SUPPLEMENTARY MATERIAL

Cannabidiol may prevent the development of congestive hepatopathy secondary to right ventricular hypertrophy associated with pulmonary hypertension in rats.

Anna Krzyżewska, Marta Baranowska-Kuczko, Anna Galicka, Irena Kasacka, Krzysztof Mińczuk, Hanna Kozłowska

Correspondence: anna.krzyzewska@umb.edu.pl

Western blot:

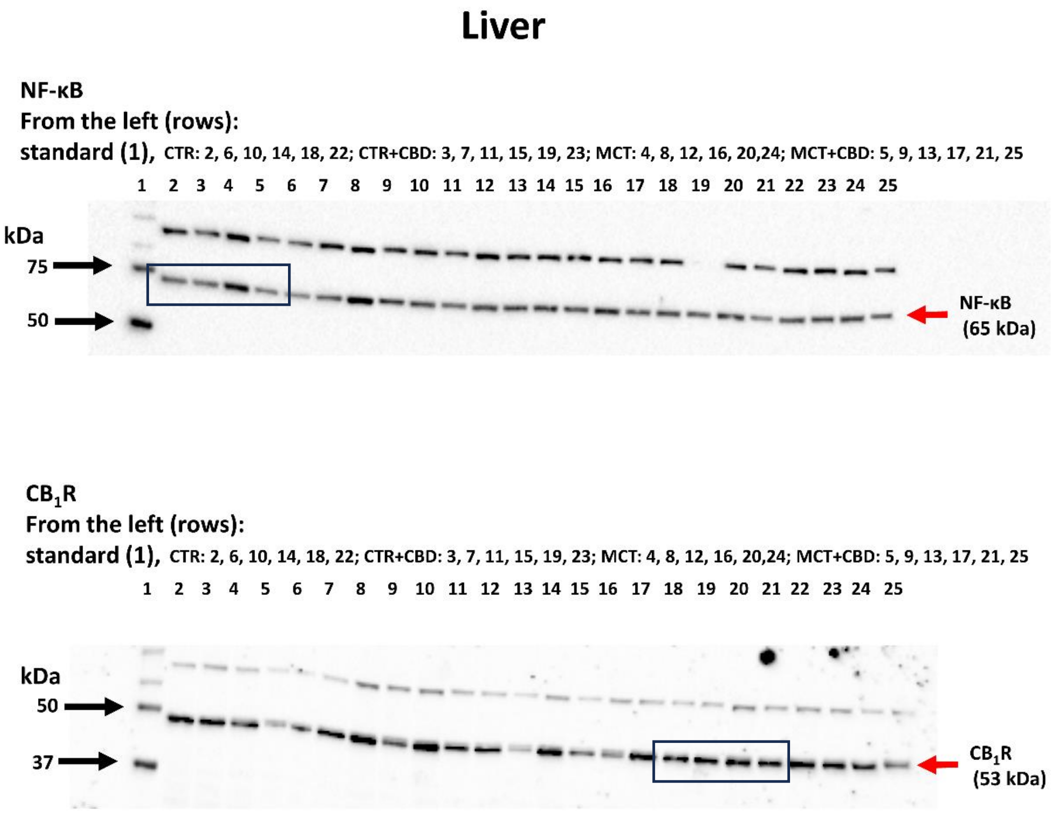

Figure S1. Uncropped western blots shown in Figure 3 and Figure 4. Abbreviations: CBD-cannabidiol; CTR-control; MCT-monocrotaline; NF-κB - nuclear factor-κappa B, CB_1R - cannabinoid receptor type 1.

The red arrows indicate bands detected with the anti- NF-κB and anti-CB_1R antibodies.

The black arrows indicate specific molecular weights obtained using the Western C standard which help identify and characterize the molecules separated in a gel

The bands highlighted with the frames are shown in the manuscript as representative images.

.

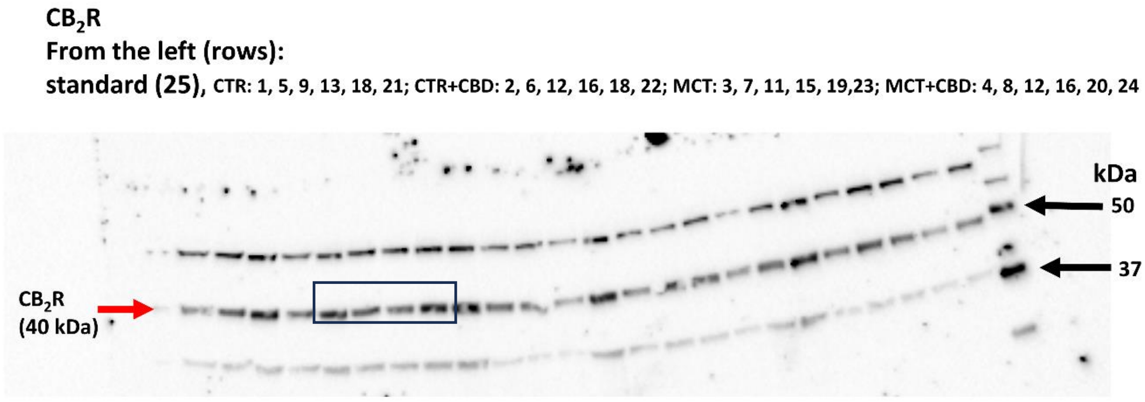

Figure S2. Uncropped western blots shown in Figure 4. Abbreviations: CBD- cannabidiol; CTR-control; MCT-monocrotaline; CB_2R – cannabinoid receptor type 2.

The red arrows indicate bands detected with the anti-CB_2R antibodies.

The black arrows indicate specific molecular weights obtained using the Western C standard which help identify and characterize the molecules separated in a gel.

The bands highlighted with the frames are shown in the manuscript as representative images.

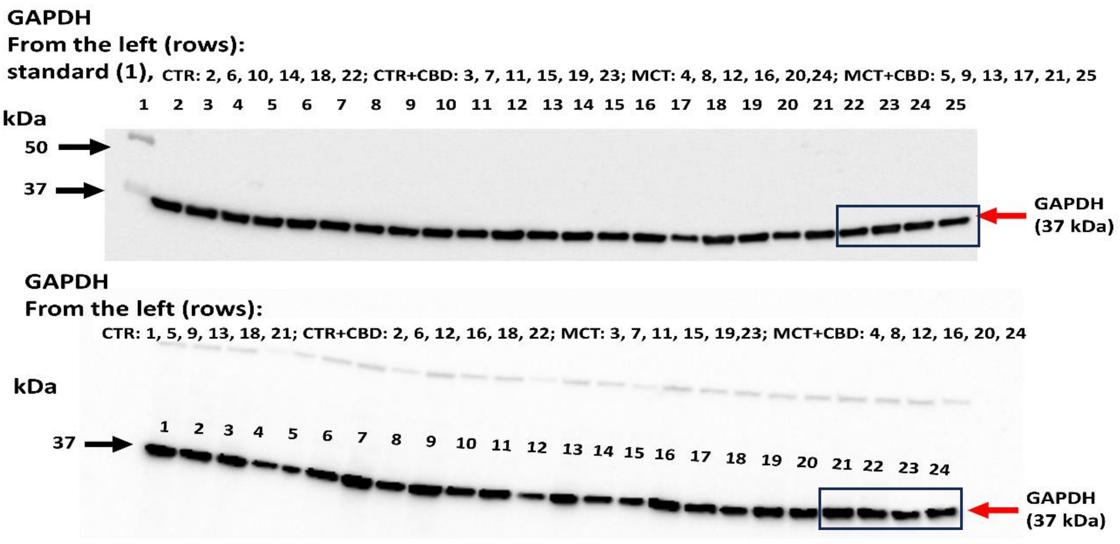

Figure S3. Uncropped western blots shown in Figure 3 and Figure 4. Abbreviations: CBD- cannabidiol; CTR-control; MCT-monocrotaline, GAPDH- Glyceraldehyde 3-Phosphate Dehydrogenase.

The red arrows indicate bands detected with the anti-GAPDH antibodies.

The black arrows indicate specific molecular weights obtained using the Western C standard which help identify and characterize the molecules separated in a gel.

The bands highlighted with the frames are shown in the manuscript as representative images.

Histology:

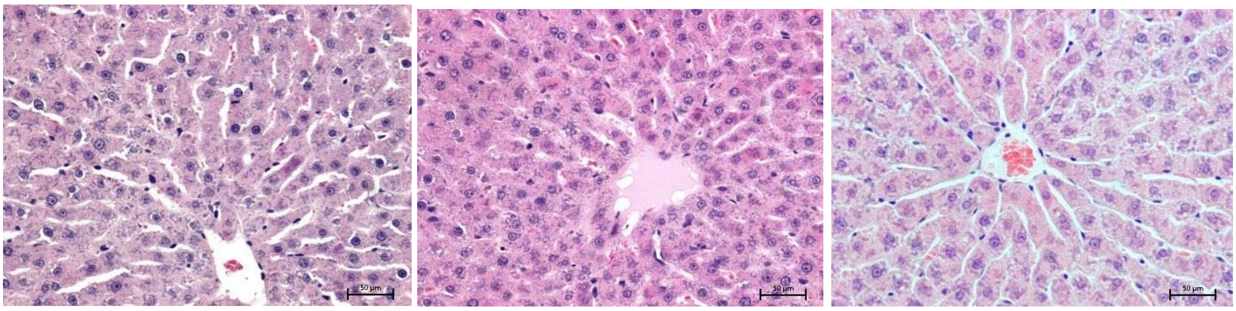

Figure S4. Example images of liver sections stained with hematoxylin and eosin from 3 different animals from control (CTR) group.

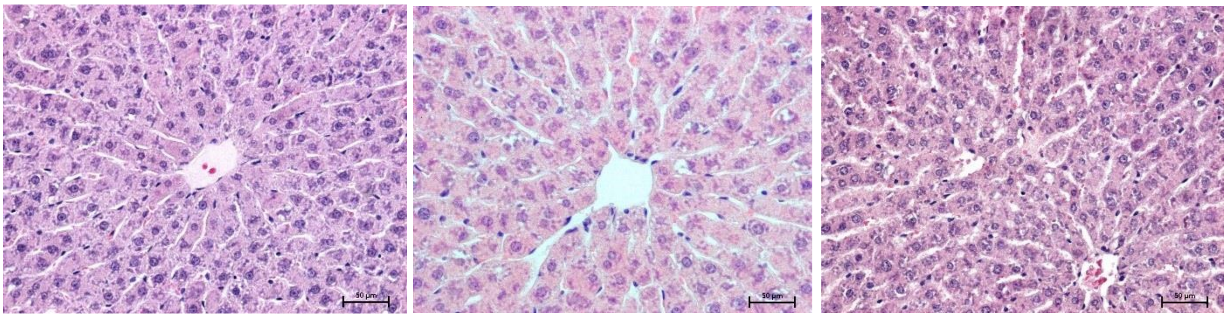

Figure S5. Example images of liver sections stained with hematoxylin and eosin from 3 different animals from control + cannabidiol (CTR + CBD) group.

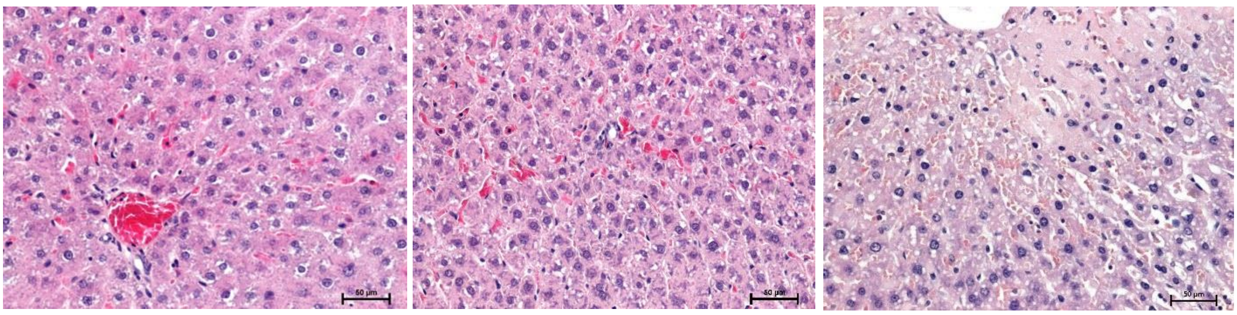

Figure S6. Example images of liver sections stained with hematoxylin and eosin from 3 different animals from monocrotaline (MCT) group.

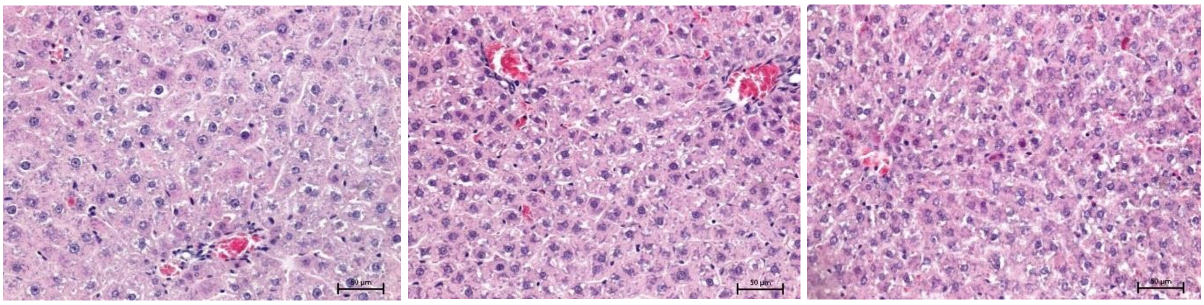

Figure S7. Example images of liver sections stained with hematoxylin and eosin from 3 different animals from monocrotaline + cannabidiol (MCT + CBD) group.
